# A genome-wide linkage study of GAW15 gene expression data

**DOI:** 10.1186/1753-6561-1-s1-s87

**Published:** 2007-12-18

**Authors:** Donghui Kan, Richard Cooper, Xiaofeng Zhu

**Affiliations:** 1 Department of Preventive Medicine and Epidemiology, Loyola University Medical Center, Maywood, Illinois 60153, USA; 2 Department of Epidemiology and Biostatistics, Case Western Reserve University School of Medicine, Wolstein Research Building, 2103 Cornell Road, Cleveland, Ohio 44106-7281, USA

## Abstract

**Background:**

Recently, gene expression levels have been shown to demonstrate familial aggregation, suggesting a direct role of heritable DNA variation. We studied the gene expression levels in lymphoblastoid cells of the Centre d'Etude du Polymorphisme Humain Utah families made available to Genetic Analysis Workshop 15 (GAW15), using genome-wide linkage analyses.

**Methods:**

Heritability was estimated for the expression levels of each individual phenotype. Genome wide linkage analysis was then performed using the 2819 SNPs for the expression levels of all the genes.

**Results:**

Heritability exceeded 0.21 for 50% of the expressed phenotypes. Genome-wide linkage analysis demonstrated that 19 of them reached significance after correcting for multiple comparisons, only 4 of which were reported previously. We did not identify any hot spots of transcriptional regulation when assuming LOD score > 5.3 for significant linkage evidence.

**Conclusion:**

Our analysis suggests that inconsistent results in comparison to the previous report may be due to the different approaches, phenotype transformation, and different pedigree data used in the analyses.

## Background

Genetic diseases are the ultimate manifestation of pathological genetic variation, although under some circumstances they may also reflect the influence of environmental factors. Gene expression at the transcript level (i.e., the "gene expression phenotype") is considered an intermediate stage between DNA sequence variation and complex traits. Recently, Cheung et al. [1] studied variation in human gene expression across the genome by comparing variation among unrelated individuals, among siblings within families, and between monozygotic twins. They found significant evidence for familial aggregation of gene expression phenotypes, suggesting a contribution from germ line genetic variation. The same group also performed genome-wide linkage analysis for expression levels of 3554 genes in 14 large Centre d'Etude du Polymorphisme Humain (CEPH) Utah families by genotyping 2756 autosomal single-nucleotide polymorphisms (SNPs). They identified significant linkage evidence for a large proportion of the expression phenotypes, further supporting a role for DNA sequence variation on these phenotypes. Furthermore, they identified regions, designated hot spots of transcriptional regulation, with significant linkage to several expression phenotypes [2]. We studied the same expression data made available to Genetic Analysis Workshop 15 (GAW15), using the variance-components method implemented in Merlin [3] in order to compare to the results obtained in the original report obtained with SIBPAL in S.A.G.E. [4]. The rationale for this comparison is that the variance-components approach may be more powerful than SIBPAL when a phenotype is normally distributed, but although SIBPAL is robust to the normality assumption, the variance-components approach is not.

## Methods

The human gene expression data in lymphoblastoid cells included 14 three-generation CEPH Utah families. The expression levels of 3554 of the 8500 genes tested were available for GAW15. In addition, 2819 autosomal SNPs were genotyped and provided by GAW15. The linkage map of the SNPs was calculated based on the deCode map using interpolation (Kong et al. [5]). We analyzed 3354 expression phenotypes after excluding SNPs on the X chromosome and those with gene locations that we were unable to locate.

### Statistical analysis

We implemented the software SOLAR to calculate heritability for each expression phenotype under the assumption of a polygenic model [6]. Genome-wide linkage analysis for each expression phenotype was then performed using the multipoint variance-components method as implemented in the software package Merlin. The variance-components method decomposes the total variance into the additive effect of a quantitative trait locus (QTL), polygenic effects, and random environmental effects. The likelihood ratio test was applied to test the null hypothesis of no additive genetic variance due to the QTL. We also performed linkage analysis using SIBPAL with w4 option for some phenotypes for comparison [4]. Because it was not our goal to address or evaluate corrections for multiple testing, in the spirit of a GAW analysis, despite the large number of tests performed, we present only point-wise test results here.

## Results

**Figure 1:**
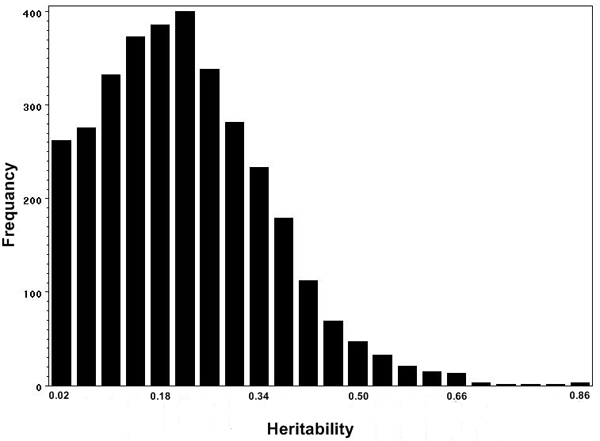
The heritability distribution of 3354 gene expression phenotypes.

Figure 1 presents the distribution of heritability of 3354 expression phenotypes. The range of heritability is from 0 to 0.87 with an average of 0.22, suggesting a modest amount of genetic contribution to the expression level. The heritability distribution did not show clustering in chromosome regions, suggesting the inheritable expression phenotypes are randomly distributed across the genome.

**Figure 2:**
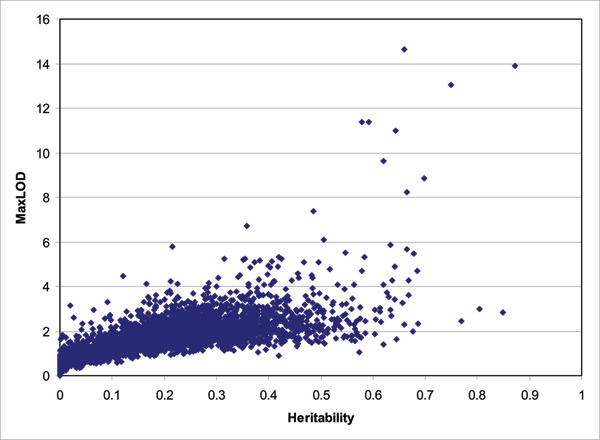
LOD scores vs. heritability.

We next performed multipoint linkage analysis using the variance-components approach implemented in Merlin. We observed 197 genome scans with LOD scores > 3.3 among the 3354 genome scans. In this report we used the criterion of a LOD score = 3.3 to correspond to a false-positive rate of ~0.05 for a genome-wide linkage analysis of one trait [7], however we acknowledge that a better approach might be through simulations. We expected 168 genome scans to have LOD score over 3.3 by chance among the 3354 scans, i.e., relatively fewer than we observed. We identified 19 expression phenotypes that reached genome-wide significance after correction for 3354 tests (LOD > 5.3) according to Morley et al. [2] and these genes are listed in Table 1. However, only 4 of them overlapped with the set of phenotypes with the strongest evidence of linkage found by Morley et al. [2], who used SIBPAL in their analysis. Given the potential concern that the inconsistencies were due to the different analysis methods, genetic maps, or phenotype transformation, we reanalyzed these 19 gene expression phenotypes using SIBPAL. Ten of the 19 gene expression phenotypes had *p*-values less than 10^-10^, suggesting these two approaches did contribute the difference of the results. Because SIBPAL is robust to the trait normality assumption [8] and the results of Morley et al. used the log transformation of the phenotypes, we also performed linkage analysis for the log transformation of the 19 gene expression phenotypes using the two methods. The LOD score of *UGT2B17 *dropped substantial using Merlin and a similar change was also observed using SIBPAL. The expression of *UGT2B17 *had a bimodal distribution before the log transformation and was skewed after the log transformation, which may explain the difference. We also observed substantial differences of linkage evidence for expression of *PYGB *and *TMED10 *when analyzed by Merlin and SIBPAL. However, we did not observe any substantial departure from a normal distribution for these two expression phenotypes either before or after the log transformation, suggesting SIBPAL may be less powerful than the variance-components method when a trait is normally distributed. The range of heritability for these expression phenotypes is between 0.22 and 0.87. The correlation between the maximum LOD score and heritability is 0.64 (Fig. 2). The correlation remains large (0.54) when we limited to the LOD scores with heritability less than 0.1.

**Table 1:** 19 gene expression phenotypes with genome-wide significant linkage evidence after correcting for multiple tests

		Merlin VC LOD		Linkage peak position			SIBPAL linkage	
					
Gene^a^	Location	log_2_-transformed	not log_2_-transformed	Chr	position (cM)	*h*^2^	log_2_-transformed	not log_2_-transformed
LOC388796	20q11	14.65	13.92	20	59.3	0.66	2.9 × 10^-13^	7.4 × 10^-13^
**ZP3**	7q11	13.91	13.34	7	84.5	0.87	1.1 × 10^-16^	4.4 × 10^-14^
**CHI3L2**	1p13	13.06	12.72	1	119.2	0.75	<10^-16^	<10^-16^
LRAP	5q15	11.38	8.51	5	105	0.58	<10^-16^	4.4 × 10^-16^
**DDX17**	22q13	11.36	10.3	22	49.8	0.59	3.0 × 10^-11^	4.4 × 10^-12^
PSPH	7p15	10.99	8.74	7	78.7	0.64	5.0 × 10^-9^	5.2 × 10^-5^
UGT2B17	4q13	9.62	3.43	4	74.8	0.62	2.7 × 10^-5^	0.03
HLA-DPB1	6p21	8.87	8.8	6	49.7	0.7	5.0 × 10^-6^	3.2 × 10^-5^
ITGB1BP1	2p25	8.25	8.31	2	25.5	0.66	4.4 × 10^-11^	2.1 × 10^-10^
CSTB	21q22	7.39	7.54	21	85.4	0.49	7.4 × 10^-10^	7.0 × 10^-9^
PPAT	4q12	6.72	6.65	4	66.7	0.36	2.8 × 10^-8^	4.8 × 10^-8^
IRF5	7q32	6.08	6.32	7	124.2	0.51	3.1 × 10^-15^	1.8 × 10^-14^
PEX6	6p21	5.86	5.67	6	56.4	0.63	2.1 × 10^-5^	9.2 × 10^-7^
RPL31	2q11	5.77	5.87	2	116.3	0.22	5.2 × 10^-11^	4.9 × 10^-14^
PYGB	20p11	5.66	3.96	4	55.6	0.67	0.085	0.38
**HSD17B12**	11p11	5.51	5.49	11	50.6	0.55	1.1 × 10^-10^	6.7 × 10^-16^
GSTM1	1p13	5.49	5.14	1	118.3	0.68	7.8 × 10^-7^	5.0 × 10^-5^
GSTM2	1p13	5.31	5.15	1	119.16	0.42	3.7 × 10^-6^	1.5 × 10^-5^
TMED10	14q24	5.31	5.27	4	56.3	0.58	0.12	0.08

^a ^Genes in bold were also reported by Morley et al. [2].

We next examined how many genes fall in the 1-LOD drop linkage region of its corresponding expression phenotype. The average width of the 1-LOD drop linkage region was 8.8 cM. Among the 197 regions with LOD scores > 3.3, only five genes fell in the 1-LOD drop region of the corresponding gene expression phenotype, indicating that the majority of the expression phenotypes are regulated by other genes. Morley et al. identified several master regulators of expression phenotypes through use of linkage evidence. We performed similar analyses by dividing the autosomal genome into 331 windows of 8.8-cM intervals and counted the number of linkage peaks falling in each window among the 197 linkage peaks. We identified five windows with over five hits and these windows are presented in Table 2. We calculated the probability of observing five or more hits per window using the same method described in Morley et al., and the probability is less than 0.00038. When the critical LOD score was increased to 4.0, we observed 81 linkage peaks and two windows with over four hits. The probability of four or more hits per window is less than 0.00012, assuming 81 linkage peaks randomly distributed. This suggested the possible existence of master regulators of transcription. We observed three hits in the hot spot region on chromosome 20 reported by Morley et al. when using the LOD > 3.3. However, the hot spot on chromosome 14 observed by Morley et al. [2] was not represented in our analysis. We did not observe any hot spots when the critical LOD score was increased to 5.3.

**Table 2:** Hotspots of transcriptional regulation

Chromosome	No. of hits	Window region (cM)
2	5	79–88
3	10	18–27
4	5	53–62
11	12	88–97
22	5	18–26

## Discussion

Gene expression phenotypes offer important insight into naturally occurring variation and might represent intermediate phenotypes between some genetic diseases and DNA variation. The genetic contribution to expression phenotypes has been studied in species from yeast to human [1,8,9]). Linkage evidence for a large proportion of the human expression phenotypes has been detected using the CEPH Utah family by Morley et al. [2]. Morley et al. also identified many hot spots of transcriptional regulation. Our heritability analysis using this data set also suggested that genetics has a modest influence on gene expression phenotypes. Overall, therefore, our results are consistent with the report by Morley et al. [2]. However, differences also appear between the two reports. Among the 13 expression phenotypes with the strongest linkage evidence reported by Morley et al., only four are present in our analysis.

Further analyses suggested several factors that might contribute to the inconsistencies, as summarized below. 1) We used different analysis approaches. In our multipoint genome-wide linkage analysis we used the variance-components approach implemented in Merlin while Morley et al. applied SIBPAL, which is robust to the normality assumption [8]. Using the exact same data for the 19 gene expression phenotypes we still obtained different conclusions regarding linkage for 10 expression phenotypes. One potential reason may be the different power for the two approaches when a trait satisfies the assumption normality. 2) The phenotype transformation may also contribute. For example, after log transformation, the linkage evidence of expression of *UGT2B17 *and *PYGB *was no longer statistically significant. 3) Morley et al. [2] did not include the data from grandparents in the analysis while we used all the family data, which may also play a role, although further confirmation is required from an analysis that does not use the grandparental data.

We failed to observe the hot spot of transcriptional regulation on chromosome 14 reported by Morley et al. [2]. This inconsistency may also be explained by the reasons we mentioned above. Also, Bastone et al. [10] reported that the evidence of hot spots of transcriptional regulation on chromosome 14 reported by Morley et al. [2] is driven by a single family, indicating that genetic heterogeneity exists in gene expression phenotypes. Wang et al. [11] performed simulation permutation analysis by including and excluding the highly correlated phenotypes, suggesting the hot spots might be artificial. Further independent studies, perhaps with larger sample size, may be required in order to identify the true biological patterns.

## Competing interests

The author(s) declare that they have no competing interests.
